# Comparison of manual sutures and laparoscopic stapler for pancreatic stump closure techniques in robotic distal pancreatectomy: a single-center experience

**DOI:** 10.1007/s00464-023-10601-0

**Published:** 2023-12-13

**Authors:** Qitao Jiang, Chao Lu, Yucheng Zhou, Qicong Zhu, Yufeng Ren, Yiping Mou, Weiwei Jin

**Affiliations:** 1 Department of Gastroenterology & Pancreatic Surgery, Zhejiang Province People’s Hospital, Hangzhou, 310000 Zhejiang People’s Republic of China; 2 Department of Medical Oncology, Zhejiang Province People’s Hospital, Hangzhou, 310000 Zhejiang People’s Republic of China; 3 Department of Surgery, Bengbu Medical College, Bengbu, 233030 Anhui People’s Republic of China

**Keywords:** Postoperative pancreatic fistula, Robotic distal pancreatectomy, Pancreatic stump closure techniques, Robotic surgery

## Abstract

**Background:**

Postoperative pancreatic fistulas (POPFs) are prevalent and major postoperative complications of distal pancreatectomy (DP). There are numerous ways to manage the pancreatic stump. However, no single approach has been shown to be consistently superior. Moreover, the potential role of robotic systems in reducing POPFs has received little attention.

**Methods:**

The clinical data of 119 patients who had consecutively received robotic distal pancreatectomy between January 2019 and December 2022 were retrospectively analyzed. Patients were divided into two groups according to the method of handling the pancreatic stump. The attributes of the patients and the variables during the perioperative period were compared.

**Results:**

The analysis included 72 manual sutures and 47 stapler procedures. The manual suture group had a shorter operative time (removing installation time) than the stapler group (125.25 ± 63.04 min vs 153.30 ± 62.03 min, *p* = 0.019). Additionally, the manual suture group had lower estimated blood loss (50 mL vs 100 mL, *p* = 0.009) and a shorter postoperative hospital stay. There were no significant differences in the incidence of clinically relevant POPFs between the two groups (18.1% vs 23.4%, *P* > 0.05). No perioperative death occurred in either group.

**Conclusion:**

The manual suturing technique was shown to have an incidence of POPFs similar to the stapler technique in robotic distal pancreatectomy and to be safe and feasible.

**Graphical Abstract:**

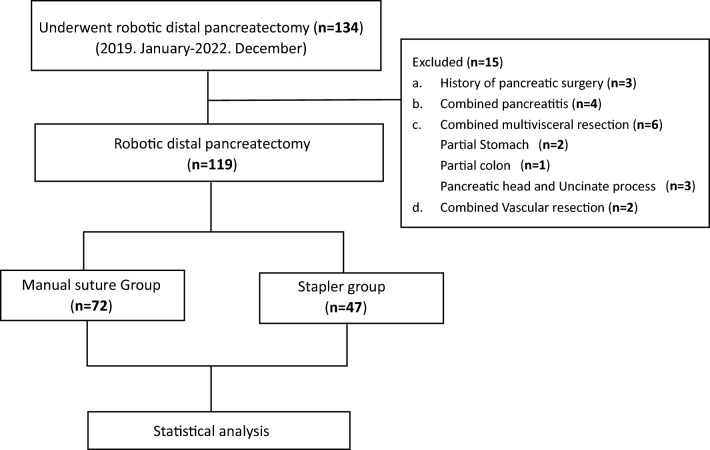

**Supplementary Information:**

The online version contains supplementary material available at 10.1007/s00464-023-10601-0.

Distal pancreatectomy is the classic procedure for treating pancreatic body and tail cancers. Postoperative pancreatic fistulas are prevalent and major complications after DP surgery and the most urgent problem to be solved following this procedure. The rate of POPFs can reach 28.6%, which prolongs the hospital stay and increases medical expenses [1–3]. Treatment of the pancreatic stump is an important factor affecting POPFs. The number of therapy options for pancreatic stumps has increased over the past few years, but no single method has yet been deemed optimal [4]. Effective management of pancreatic stumps to prevent POPFs remains a challenge.

The da Vinci surgical robotic system is the most recent advanced minimally invasive approach for distal pancreatectomy. The feasibility and safety of robotic DP (RDP) have been confirmed by previously published studies, and RDP has demonstrated favorable results regarding transfer rate and spleen preservation rate [5–8]. These advantages have been initially confirmed. Moreover, owing to its 10 × magnification in 3D imaging, 540° moving range of surgical equipment, and improved flexibility in complex procedures [9], robotics appears to be crucial for decreasing POPF rates during the handling of pancreatic stumps.

In the ever-expanding realm of RDP procedures, diverse research institutions employ a myriad of techniques to address the management of stumps. However, none of these approaches has demonstrated unwavering superiority over the others. This study aimed to answer the question, how do the postoperative outcomes of individuals undergoing RDP compare with each other and how do the outcomes of different strategies for the management of pancreatic stumps differ? We hope that this study will provide our center’s clinical experience for individualized pancreatic stump management.

## Materials and methods

**Fig. 1:**
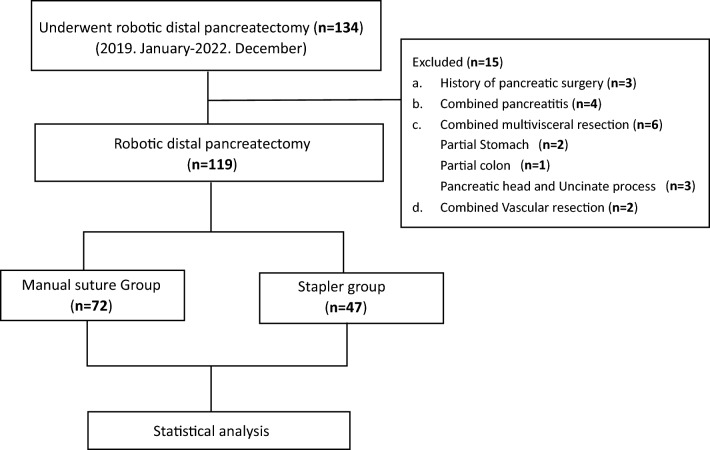
Flowchart of the scheme for different methods of the closure of pancreatic stump during robotic distal pancreatectomy (RDP)

The clinical records who had undergone RDP at Zhejiang Provincial People’s Hospital between January 2019 and December 2022 were retrospectively researched. Patients who had undergone prior pancreatic surgery, those with combined pancreatitis, and those who had undergone extended resection during surgery (including combined multivisceral and vascular resection) were excluded from this study. Ultimately, 119 patients who met the specified inclusion and exclusion criteria were selected for this study, as depicted in Fig. 1. Two groups of patients were defined based on the technique used to manage the pancreatic stump: manipulating the pancreatic stump with hand-sewn sutures (manual suture group) and using a traditional laparoscopic stapler (stapler group). Two surgeons with extensive experience in open DP and laparoscopic DP (LDP), who had successfully completed a series of robotic hepatobiliary and pancreatic surgeries, and who were from the same department performed the operations. Abdominal imaging procedures were performed on all patients, including enhanced computed tomography (CT) scans and magnetic resonance imaging (MRI).

The Ethics Committee of Zhejiang Provincial People’s Hospital approved this study (QT2023107), and all patients provided written informed consent. Additionally, the study was conducted according to the STROBE reporting guidelines.

### Data collection

Patients’ preoperative characteristics extracted from the hospital database included age, gender, American Society of Anesthesiologists (ASA) score, albumin, and body mass index (BMI). Based on preoperative radiologic scans (CT and/or MRI), the thickness of the pancreatic parenchyma in the neck of the pancreas was assessed. Surgical variables included operative time, technique used to manage the pancreatic stump, pancreatic texture, length of the resected pancreas, and estimated blood loss. Postoperative consequences comprised postoperative hospital stay duration, unexpected reoperation, readmission, mortality, and associated postoperative complications (the grades of postoperative complications were recorded according to the Clavien–Dindo classification [10, 11]), as well as patients’ pathology results.

According to the definition and grading system of POPFs released by the 2016‐Revised International Study Group on Pancreatic Surgery classification [12], POPFs are diagnosed as an amylase value in the drainage fluid that is higher than three times the upper limit of normal on or after the third day following the operation. Simultaneously, it has a certain clinical impact, and active clinical treatment is required. Further classification irrespective of the clinical course as a biochemical fistula, Grade B is divided into the following situations: (1) abdominal drainage tube indwelling time > 3 weeks; (2) the clinical treatment plan changed because of the pancreatic fistula; (3) pancreatic fistulae require percutaneous or endoscopic puncture drainage; (4) pancreatic fistula-related bleeding requires angiographic intervention to stop bleeding; and (5) a pancreatic fistula leads to infection, but no organ failure. Grade C tumors require surgical treatment and lead to organ failure or death.

The preoperative, intraoperative, and postoperative data of the two groups were analyzed and contrasted.

### Technical notes

**Fig. 2:**
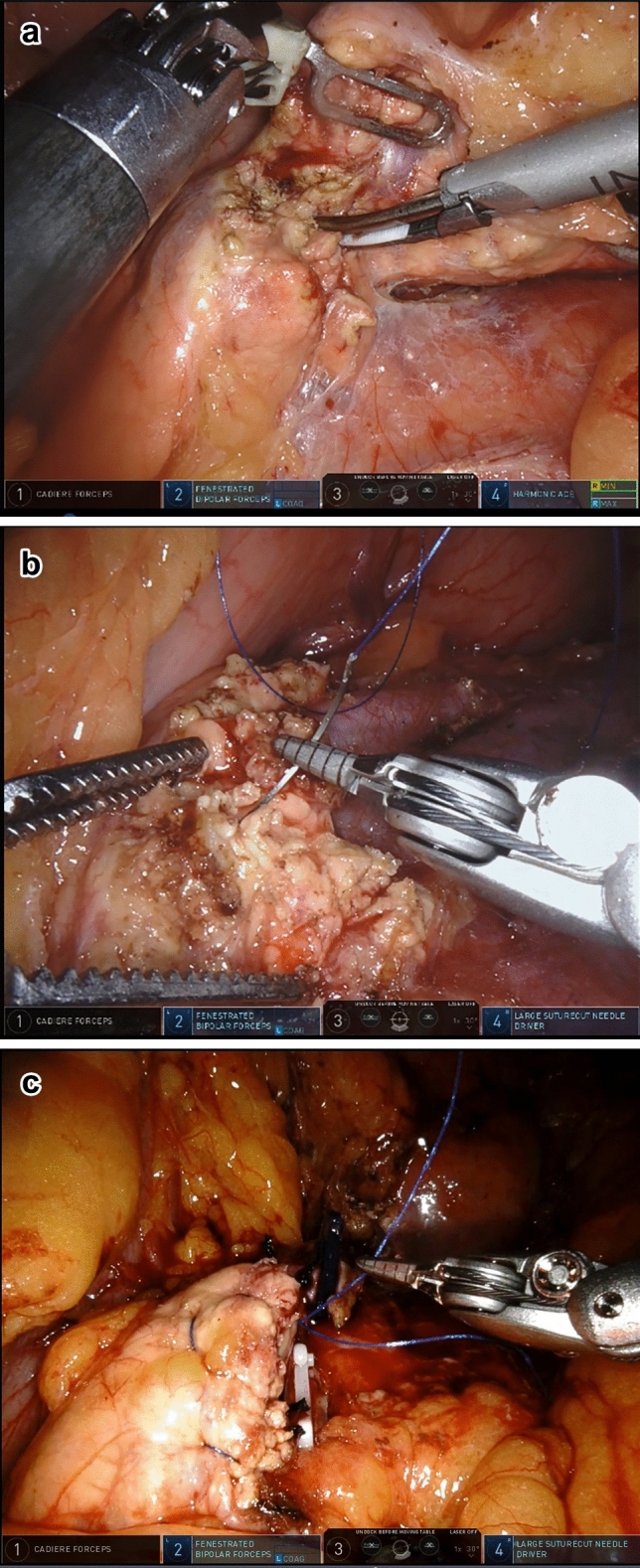
The technique of manual suture. **a** Using ultrasonic coagulating shears to separate the pancreas. **b** Ligating the main pancreatic duct. **c** Closuring pancreatic stump in a fish-mouth manner

**Fig. 3:**
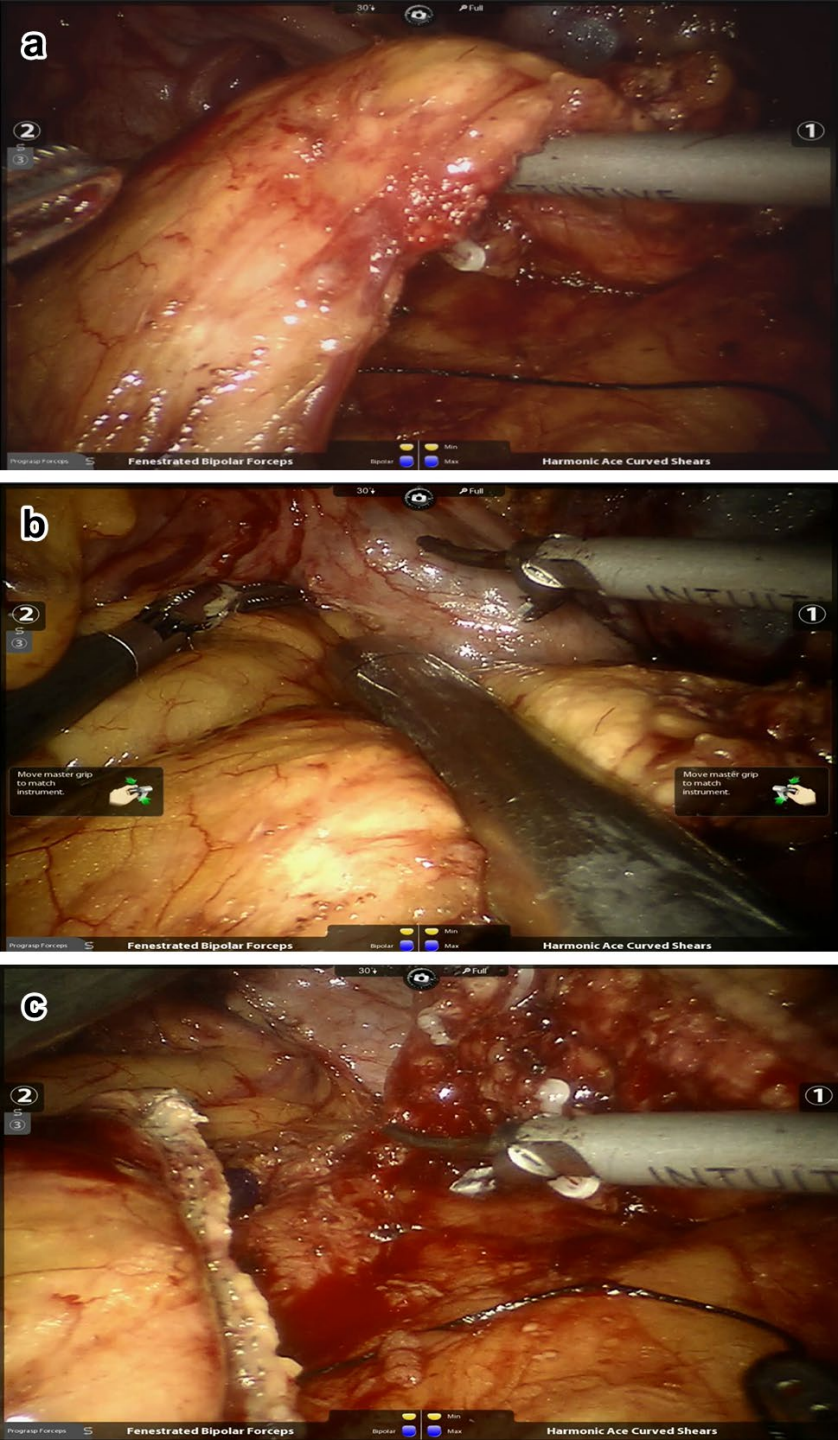
The technique of stapler. **a**, Running through the posterior pancreatic tunnel. **b**, Using the suitable cartridge to clamp and compress the pancreas. **c**, Cutting off the pancreatic tissue and closing the stump

The procedure for DP has been reported in detail [13]. The da Vinci Si or Xi system (Intuitive Surgical Inc., CA) was employed in all procedures. A technique consisting of five ports was utilized. Depending on the surgeon’s choice, the remaining pancreatic stump was managed using two distinct approaches. (Figs. 2 and 3).

1. Through the pancreas’ upper and lower margins, an ultrasonic coagulating shears is used to separate the pancreas from the proximal end 2 cm away from the lesion. The pancreas section is shaped like a fish mouth. Next, the main pancreatic duct is tied off and the pancreatic stump is stitched shut using intermittent “U” sutures with four or five 5/0 prolene sutures (manual suture group).
2. We regularly establish an assistant port on the left midclavicular line at the umbilicus level, which accommodated a 12-mm Trocar. The assistant places the laparoscopic stapler through this port to complete the operation. After freeing the lower edge of the distal pancreas from the tail of the pancreas, the stapler is run through the posterior pancreatic tunnel. The parenchyma is transected with a laparoscopic stapler (ECHELON FLEX™ Powered Plus Articulating Endoscopic Linear Cutters, PSEE45A). According to the surgeon’s perception of thickness, we may choose the white cartridge (ECHELON ENDOPATH™ Endoscopic Linear Cutter Reloads, GST45W), which allows an opening staple height of 2.6 mm and a post-fired height of 1 mm. If the pancreatic parenchyma is thicker than 3 mm, the blue cartridge (GST45B) might need to be used, in which case the height will change from 3.6 to 1.5-mm post-firing. Re-firing compression is routinely performed (stapler group).

### Statistical analysis

Quantitative data were tested for normal distribution using the Kolmogorov–Smirnov test and for homogeneity of variance using an appropriate test. Data that followed a normal distribution were expressed as mean ± standard deviation (χ ± s), and statistical analysis was performed using the Student’s t test. The non-normally distributed assessment data are indicated as median M (P25–P75), and the Mann–Whitney U test was utilized for statistical analysis. Categorical variables are presented as numbers of cases (percentage), and intergroup comparisons were performed using the χ^2^ test or Fisher’s exact probability method. *p* < 0.05 was regarded as significant. All statistical analyses were performed using IBM SPSS software (Version 25.0, IBM Corp., Armonk, NY, USA).

## Results

Among the 119 selected patients, 72 and 47 patients were included in the manual suture and stapler groups, respectively. No significant variations were found between the two groups in terms of age, sex, albumin, ASA score, BMI, or tumor type (*p* > 0.05). Demographic details of the patients are described in Tables 1 and 2.

**Table 1:** Patient characteristics

	Manual suture (*n* = 72)	Stapler (*n* = 47)	p
Gender (male/female)	32/40	25/22	0.350
Age (year)	58.67 ± 15.18	54.28 ± 16.52	0.139
BMI (kg/m^2^)	22.78 ± 3.40	22.34 ± 2.94	0.468
Albumin (g/L)	40.06 ± 3.79	40.54 ± 4.71	0.539
ASA			0.051
I–II	49 (68.1%)	40 (85.1%)	
III–IV	23 (31.9%)	7 (14.9%)	
Thickness of the pancreas (cm)	2.88 ± 0.65	3.10 ± 0.60	0.068

*BMI* body mass index, *ASA* the American Society of Anesthesiologists

**Table 2:** Pathologic data

	Manual suture (*n* = 72) (%)	Stapler (*n* = 47) (%)	*p*
PDAC	30 (41.7)	16 (34.0)	0.171
IPMN	4 (5.6)	3 (6.4)	
Serous cystic neoplasm	8 (11.1)	6 (12.8)	
Mucinous cystic neoplasm	7 (9.7)	2 (4.3)	
Solid pseudopapillary neoplasm	6 (8.3)	7 (14.9)	
Neuroendocrine tumor	2 (2.8)	7 (14.9)	
Chronic pancreatitis / Pseudocysts	4 (5.6)	4 (8.5)	
Ectopic spleen	4 (5.6)	0 (0)	
Other benign lesions	3 (4.2)	0 (0)	
Other malignant lesions	4 (5.6)	2 (4.3)	

*PDAC* pancreatic ductal adenocarcinoma, *IPMN* intraductal papillary mucinous neoplasm

The operative time (removing installation time) was shown to be significantly shorter in the manual suture group than in the stapler group (125.25 ± 63.04 vs 153.30 ± 62.03 min, *p* = 0.019). The estimated blood loss was lower in the hand-sewn group than that in the stapler group (50 vs 100 mL, *p* = 0.009). The two groups had similar distributions of pancreatic texture and resected pancreatic length (Table 3).

**Table 3:** Operative variables

	Manual suture (*n* = 72)	Stapler (*n* = 47)	*p*
Operation time (min)	125.25 ± 63.04	153.30 ± 62.03	0.019
Estimated blood loss (ml)	50 (20–100)	100 (50–150)	0.009
Length of resected pancreas (cm)	8.99 ± 3.25	8.87 ± 3.45	0.838
Pancreas texture			0.549
Soft	30 (41.7%)	17 (36.2%)	
Hard	42 (58.3%)	30 (63.8%)	

Table 4 displays the postoperative outcomes. The manual suture group had a longer postoperative hospital stay than the stapler group (10 vs 11 days, *p* = 0.045). Major complications (Clavien–Dindo ≥ grade III) occurred in 11.8% (14/119) of all patients, and the rates of the main complications, including hemorrhage, reoperation, and 90-day mortality were similar in both groups (Table 4).

**Table 4:** Postoperative outcomes

	Manual suture (*n* = 72)	Stapler (*n* = 47)	*p*
Major complications (Clavien–Dindo Grade IIIa or above)	6 (8.3%)	8 (17.0%)	0.228
IIIa	6 (8.3%)	6 (12.8%)	
IIIb	0 (0)	1 (2.1%)	
IV	0 (0)	1 (2.1%)	
V	0 (0)	0 (0)	–
POPF			0.304
None	15 (20.8%)	5 (10.6%)	
Biochemical leak	44 (61.1%)	31 (66.0%)	
Grade B	13 (18.1%)	10 (21.3%)	
Grade C	0(0)	1 (2.1%)	
Reoperation	0(0)	2 (4.3%)	0.154
Postoperative hemorrhage	0(0)	3 (6.4%)	0.059
Postoperative hospital stays (d)	10 (8–12)	11 (9–15)	0.045
90-day mortality	0 (0)	0 (0)	–

*POPF* postoperative pancreatic fistula

Among 119 patients, there were 75 patients with biochemical leak, 23 patients with grade B pancreatic fistula (with an incidence of 19.3%), and only one patient with grade C pancreatic fistula, accounting for 0.84%. In the manual suture group, the rate of clinically relevant POPFs (CR-POPF; Grade B or above) was 18.1% (13/72) versus 23.4% (11/47) in the stapler group; however, the difference was not statistically significant (*p* = 0.304). Five patients in the stapler group underwent invasive treatment for POPFs, including one who underwent reoperation and four who underwent ultrasound-guided peritoneal fluid puncture and drainage. Four patients in the hand-sewn group underwent puncture and drainage.

Among the group that utilized staplers during surgery, there were three instances of postoperative bleeding. In one of these cases, the blood oozing from the wound surface of pancreatic stump, but it was effectively treated using conservative methods. The other two cases involved arterial or venous vascular hemorrhage and necessitated a subsequent surgery due to issues with circulation. The analysis of variance did not reveal any significant differences between the various groups. It is worth noting that none of the patients in the manual suture group experienced hemorrhage or required a second surgery.

## Discussion

The results of this study showed the use of both manual sutures and staplers for RDP to be acceptable and feasible, and these two techniques were equally effective in reducing the POPF rate. Robotics has gained popularity in pancreatic surgery since Melvin et al. carried out the initial RDP in 2003 [14]. Current research reports suggest that RDP has less bleeding, faster postoperative recovery, and the advantage of minimal invasiveness [8, 15–18]. Compared to LDP, spleen preservation rates are higher and transfer rates are lower in RDP. In a recent meta-analysis of 2514 RDP and 4243 LDP cases, the conversion rate and spleen preservation rate of RDP were better, and the CR-POPFs and major complications were comparable with those of LDP [19].

POPFs are the most prevalent complication of pancreatic tail resection; however, they remain unresolved, with the POPF rate for RDP having reached 24.3% [7]. A POPF is generally associated with pancreatic thickness, duct diameter, and patient factors, among which the management of the pancreatic stump is an important factor affecting POPFs [1, 20]. The selection of stump closure technique is crucial for reducing complications, such as pancreatic fistula and hemorrhage. Presently, the methods primarily include traditional manual suture, cutting closure, closure combined with hand-sewn closure, and others, such as pancreatic-intestinal anastomosis, pancreatic-gastric anastomosis, self-tissue (great omentum or ligamentum teres hepatis) wrapping, or new material covering, which are less commonly used in clinical practice. The approach varies from center to center and there is no consensus on the management of pancreatic stumps. In a multicenter study that included 2026 patients, the rate of clinical POPFs was significantly reduced by the cut-and-close method compared with manual suturing (19.1% vs 12.7%, *p* < 0.001) [1]. Concurrently, a meta-analysis that included 31 studies showed lower rates of POPFs with the stapler method (*OR* = 0.55, *p* = 0.042) [21]. However, it has also been reported that the clinical POPF rate of manual suture is lower than that of stapler closure (21% vs 55%, *p* = 0.02) [22], while the DIStal PAnCreaTectomy (DISPACT) trial [23] showed that there was no statistically significant difference in pancreatic fistula rate, mortality, or complication rates between the two techniques for dealing with pancreatic stumps.

Laparoscopic techniques have been reported in the management of pancreatic stumps. Studies have shown that in cases in which the pancreatic thickness < 12 mm, the closure suture is better than the manual suture in reducing POPF rates [24]. However, there are few studies on robotics based on this aspect. Nonetheless, robotic systems appear to be useful for managing pancreatic stumps with advantages in reducing POPFs. The specific performances are as follows: on one hand, based on thorough visualization, the robotic system can perform precise surgical operations on the lesion and effectively reduce bleeding compared with traditional surgery. On the other hand, the robotic system can achieve a more precise positioning ability than the human eye can and is capable of filtering the shaking of the operator’s hand, making the suture finer and more precise to reduce the risk of POPFs [25, 26]. Our experience is that delicate operations such as hemostasis and suturing are performed more smoothly in robotic surgery than in laparoscopic surgery. Although no significant differences were shown among the postoperative outcomes of the two methods in our study, the advantages of robotics surgery in dealing with the pancreatic stump were affirmed.

When considering various factors, one should take into account the role of surgical doctors. Firstly, the pancreatic parenchyma at the neck varies in thickness, and the morphology of the pancreas is irregular. However, the closure of the residual end using staplers is relatively mechanical and cannot allow for individualized stitching. Conversely, manual suturing can achieve a “perfect closure” of the pancreas by adjusting the angles of needle insertion and exit. Secondly, the closure of the main pancreatic duct is crucial in the management of POPF. Compared to the stapler procedure, manual suturing allows for independent suturing of the main pancreatic duct, reducing the occurrence rate of POPFs. Indeed, it should be acknowledged that in certain cases, tumors that are located near the neck of the pancreas or are of significant size can make it difficult to pass through the posterior pancreatic tunnel or to place the staplers. In such situations, it may be necessary to first divide the pancreas and then proceed with closure. We believe that manual suturing may have advantages over stapler closure in certain cases. While there may not be significant differences in outcomes, there seems to be a trend in favor of manual suturing.

In this study, 119 patients underwent RDP, and the CR-POPF rate was 20.2%, which is comparable to the results of other studies. In other reports, the operative time in the hand-sewn group was greater than that in the stapler group [27], contrary to the results of this study. This may be related to the operator’s surgical technique and whether he has passed the learning curve, as the closure technique is an early approach for dealing with the pancreatic stump in our center.

Pancreatic surgery is well known to be more difficult and has a longer learning curve than other procedures. Robotic technology simplifies laparoscopic surgery, allowing surgeons with no laparoscopy experience to overcome the learning curve. With the improvement of surgical techniques and structured training programs, operation time will no longer be a limiting factor for manual suturing techniques, and this may have an impact on subsequent postoperative outcomes [28–30].

This study has several limitations, including its retrospective design, inherent selection bias, and small sample size. In addition, the technology selection at our center during different periods may also cause deviations. We aim to increase the sample size in subsequent studies and exclude any potential biases through propensity score matching. In addition, in this study, a conventional laparoscopic closure device was used instead of SureForm staplers (DA Vinci Platform), which may result in different outcomes. Prospective studies are needed to substantiate this viewpoint.

## Conclusion

The results of this study showed the use of manual sutures to be safe and feasible for RDP, and the two techniques appeared to be equivalent regarding reduced POPF rate. Owing to robotic technology that facilitates accurate dissection and fine manipulation of sutures in DP, manual suturing had a shorter operative time, less bleeding, and shorter postoperative hospital stays.

### Supplementary Information

Below is the link to the electronic supplementary material.

Supplementary file1 (DOC 98 kb)

## Data Availability

The data that support the findings of this study are available from the corresponding author upon reasonable request.
